# Surface Finish Analysis in Single Point Incremental Sheet Forming of Rib-Stiffened 2024-T3 and 7075-T6 Alclad Aluminium Alloy Panels

**DOI:** 10.3390/ma14071640

**Published:** 2021-03-27

**Authors:** Tomasz Trzepieciński, Andrzej Kubit, Andrzej Dzierwa, Bogdan Krasowski, Wojciech Jurczak

**Affiliations:** 1 Department of Materials Forming and Processing, Rzeszow University of Technology, al. Powst. Warszawy 8, 35-959 Rzeszów, Poland; 2 Department of Manufacturing and Production Engineering, Rzeszow University of Technology, al. Powst. Warszawy 8, 35-959 Rzeszów, Poland; akubit@prz.edu.pl; 3 Department of Mechanics and Machine Building, Carpatian State School in Krosno, ul. Żwirki i Wigury 9A, 38-400 Krosno, Poland; bogdan.krasowski@kpu.krosno.pl; 4 Faculty of Mechanical and Electrical Engineering, Polish Naval Academy, ul. Śmidowicza 69, 81-127 Gdynia, Poland; w.jurczak@amw.gdynia.pl

**Keywords:** 2024-T3, aluminium alloy, incremental sheet forming, load-carrying capacity, surface finish, surface roughness

## Abstract

The article presents the results of the analysis of the interactions between the single point incremental forming (SPIF) process parameters and the main roughness parameters of stiffened ribs fabricated in Alclad aluminium alloy panels. EN AW-7075-T6 and EN AW-2024-T3 Alclad aluminium alloy sheets were used as the research material. Panels with longitudinal ribs were produced with different values of incremental vertical step size and tool rotational speed. Alclad is formed of high-purity aluminium surface layers metallurgically bonded to aluminium alloy core material. The quality of the surface roughness and unbroken Alclad are key problems in SPIF of Alclad sheets destined for aerospace applications. The interactions between the SPIF process parameters and the main roughness parameters of the stiffened ribs were determined. The influence of forming parameters on average roughness *Sa* and the 10-point peak–valley surface roughness *Sz* was determined using artificial neural networks. The greater the value of the incremental vertical step size, the more prominent the ridges found in the inner surface of stiffened ribs, especially in the case of both Alclad aluminium alloy sheets. The predictive models of ANNs for the *Sa* and the *Sz* were characterised by performance measures with R^2^ values lying between 0.657 and 0.979. A different character of change in surface roughness was found for sheets covered with and not covered with a soft layer of technically pure aluminium. In the case of Alclad sheets, increasing the value of the incremental vertical step size increases the value of the surface roughness parameters *Sa* and *Sz*. In the case of the sheets not covered by Alclad, reduction of the tool rotational speed increases the *Sz* parameter and decreases the *Sa* parameter. An obvious increase in the *Sz* parameter was observed with an increase in the incremental vertical step size.

## 1. Introduction

Appropriate selection of the parameters of the sheet metal forming process using single point incremental forming (SPIF) is intended to ensure appropriate surface quality in terms of roughness and, at the same time, an economically justified forming time [1,2]. The basic parameters in the process are: tool rotational speed, feed rate, step size, size and shape of the tool, type of sheet metal, and tool path [3,4,5]. The lubricant also plays a very important role. The surface roughness of the specimen is the result of the influence obtained between the tool and specimen, where the lubricant plays a significant role during the forming process [6]. In order to optimise the process parameters, the Taguchi plan is often used [7,8,9]. Baruah et al. [10] conducted optimisation studies for the process of forming sheets of EN AW-5052 aluminium alloy using the Taguchi method. The results showed that effective lubrication is of the greatest importance for the formability and reduction of roughness in incremental sheet forming (ISF). Research on the influence of lubrication on surface quality was also carried out by Hussain et al. [11]. It was confirmed that the quality of the surface depends to a large extent on the properties of the lubricant and the method of its application.

One characteristic of the ISF forming process is the concentration of the deformation zone in the contact area of the tool face with the formed sheet. The size of this zone depends on the shape and size of the tool end and affects the formability of the sheet metal and its surface properties. Ham and Jeswiet [12,13] showed that a higher degree of formability is achieved with a smaller tool diameter due to the relatively large deformations concentrated in a small area of the material. Bhattacharya et al. [14] and Buff et al. [15] showed that tool rotational speed, feed rate and vertical step size have a significant impact on the formability of the ISFed sheets. Based on the experimental results, Ham and Jeswiet [12] claim that a low feed rate has a positive effect on sheet deformability. Vertical step size has a very significant influence on the formability and roughness of the shaped surface. Using the lowest possible step size values is recommended in order to obtain an appropriate surface quality [16,17,18]. Bhattacharya et al. [14] carried out research on the forming of EN AW-5052 aluminium alloy sheets and focussed on the effect of the step-down on the surface roughness of the draw piece surface. It was clearly indicated that an increase in the step size causes an increase in the surface roughness of the processed surface.

The investigations by Rubino et al. [19] and Andrade-Campos et al. [20] focussed on the strain distribution induced by the single point incremental forming of friction-stir-welded AA6082 sheets. It was found that in good-quality welds, material failure during SPIF occurs in the base material, evidencing that the mechanical properties of the welds are equal or better than those of the original material. Tucci et al. [21] developed an integrated numerical model able to simulate the manufacturing route for formed 6000 series aluminium alloy sheets welded by friction stir welding technology and finally shaped by means of a SPIF process. Thuillier et al. [22] performed the incremental forming of 6082-T6 aluminium alloy welded blanks until the fracture of truncated cones. The aim was to build an experimental database of the whole joining and forming process. Lu et al. [23] improved the surface quality of the parts by appropriately developing the tool path. A detailed review of the current state-of-the-art ISF processes in terms of their specific limitations, with discussions on the ISF process parameters and their effects on ISF processes, has been provided by Gatea et al. [24]. Rattanachan and Chungchoo [25] used the 2*k*-*p* factorial experimental design to analyse the interaction between the side overlap, step depth, tool feed rate and inner surface roughness. It was found that reducing tool rotational speed and feed rate reduced inner surface roughness.

In recent decades, artificial neural networks (ANNs) have become connections of elements called artificial neurons used to analyse complex regression problems [26,27,28]. There are many topologies of neural networks used to process the relationship between the explanatory and explained parameters [29,30]. Ambrogio et al. [31] proposed the implementation of ANN for predicting the geometrical variability of incrementally formed draw pieces. They also compared the Levenberg–Marquardt and backpropagation algorithms for predicting material failure. Kurra et al. [26] modelled the surface roughness in SPIF using ANNs and genetic programming. The optimum process parameters in SPIF were obtained and validated through these experiments, and highly satisfactory results were found with an error of less than 10%. Alsaman et al. [32] applied ANNs and the regression model to analyse the SPIF process for conical draw pieces. Najm and Paniti [4] used ANNs to predict the Ra and Rz parameters by adopting the data collected from frustum cones that were formed by SPIF. The results showed that an ANN with one argument in the output predicted the outcome sufficiently well when compared with a two-argument structure. Oraon and Sharma [33] used the model of the ANN to predict the quality of the SPIF component’s surface. The inputs were wall angle, feed rate, step size, sheet thickness, spindle speed and density of lubricant.

The research presented in the literature is mainly based on the SPIF of sheets that are not covered with a soft anticorrosion coating. The SPIF parameters should ensure the continuity of the protective layer after forming. This is especially important when forming sheets of high-strength EN AW-2024-T3 Alclad and EN AW-7075-T6 Alclad aluminium alloys commonly used in aircraft structures. The change in the thickness of the soft Alclad (technically pure aluminium) is related to the deformation of the core material and the mechanical interaction of the spherical end of the tool. Therefore, the SPIF process of Alclad sheets is different from that commonly studied in the literature, and in this paper, analysis is carried out on the change in surface roughness during the forming of rib-stiffened panels made of aluminium alloy Alclad sheets. The panels were formed using the single point incremental forming technique with a tool with a spherical end and a continuous spiral-shaped tool strategy. The load-carrying ability of rib-stiffened EN AW-2024-T3 and EN AW-7075-T6 aluminium alloy panels under axial compression has been studied in recent papers by the authors of [34,35]. In this paper, the effect of the forming parameters on the surface finish of the inner surface of stiffened ribs is studied based on experimental measurements of the surface roughness and on the artificial neural networks.

## 2. Materials and Methods

### 2.1. Material

Rib-stiffened panels were fabricated based on sheets with dimensions 160 mm × 120 mm (0.8-mm-thick EN AW-7075-T6 Alclad and 0.4-mm-thick EN AW-2024-T3 Alclad) and 160 mm × 100 mm (1-mm-thick EN AW-2024-T3). The chemical composition and the basic mechanical properties of the materials of the test sheets are shown in Table 1 and Table 2, respectively.

The tests were conducted in lubricated conditions using SAE 75W-85 gear oil supplied by Mannol (Wedel, Germany). About 5 mL of oil was used on each panel during the forming process. Basic physicochemical properties of the oil used are listed in Table 3.

### 2.2. Forming Method

**Figure 1:**
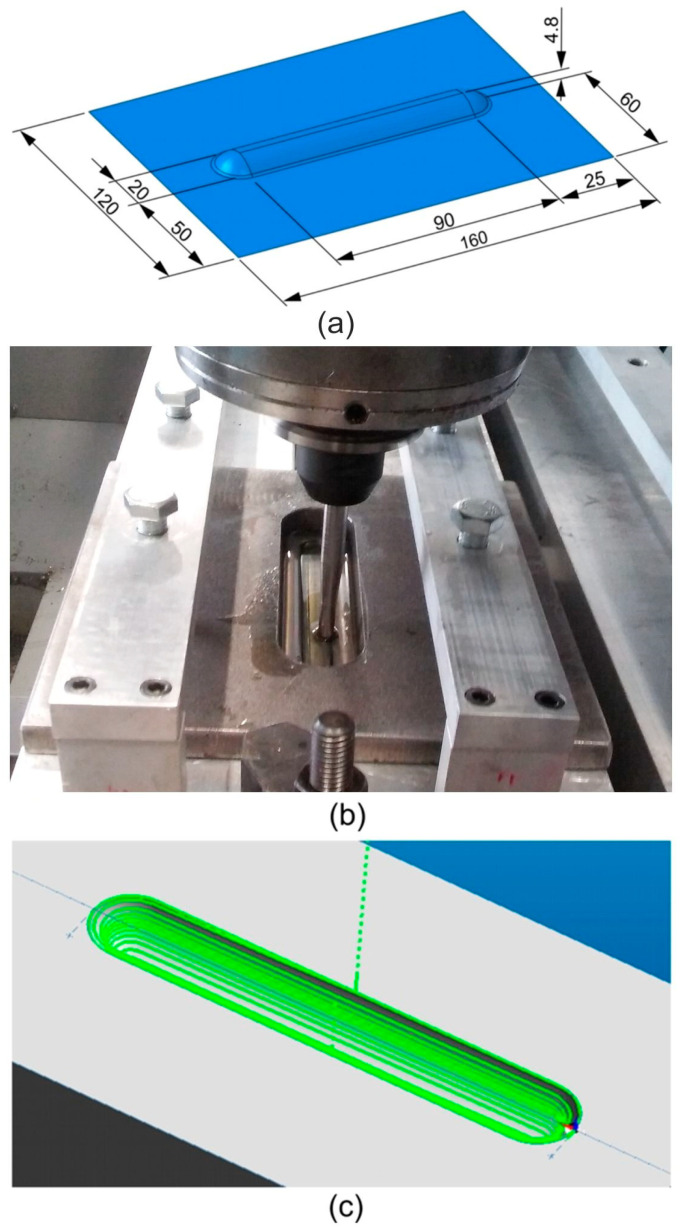
(**a**) Shape and dimensions of a rib-stiffened panel, (**b**) work-stand for single point incremental forming (SPIF) and (**c**) continuous spiral-shaped tool strategy generated in the EdgeCAM program.

The ribs, with a width of 20 mm and an inner radius of 10 mm, were formed in the middle part of the panel (Figure 1a). During the experimental tests, 7 mm diameter hemispherical, high-speed HS2-9-2 steel was used for forming.

The research was planned according to an orthogonal plan (two variable factors at three levels). A constant feed rate of 800 mm/min was used in the study. Incremental vertical step size *a_p_* and tool rotational speed *v* (Table 4) were selected as variable parameters. The same research plan was applied to all the grades of sheet metals.

The device (supporting plate and blank holder) for SPIF was mounted on the table (Figure 1b) of a numerically controlled TM-1P vertical milling machine (Hass Automation, Oxnard, CA, USA). The sheet was placed between the supporting plate and the blank holder and clamped firmly at the edges using screws. During forming, the tool moves in accordance with a path designed in the EdgeCAM program (Hexagon AB, Stockholm, Sweden). A continuous spiral-shaped toolpath (Figure 1c) was used in the investigations.

### 2.3. Analysis of Surface Roughness

**Figure 2:**
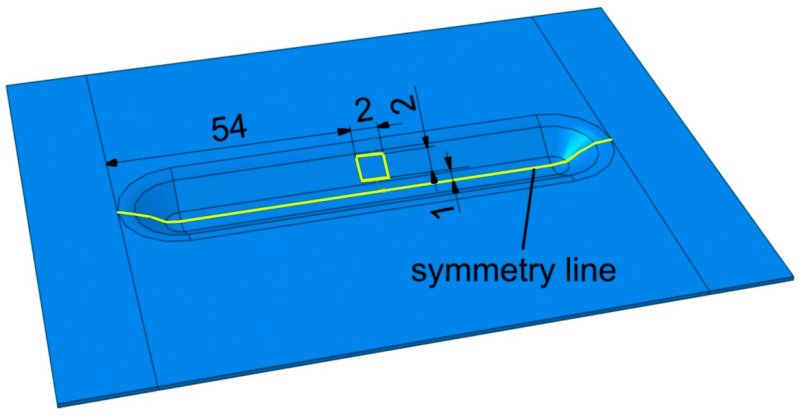
Location of measurement of the surface roughness in the inner surface of the stiffening ribs.

Scanning electron microscopy (SEM) was used to investigate wear on the inner surface of the formed ribs. Therefore, characterisation of the SEM samples was carried out on an S-3400 Phenom ProX SEM (Nanoscience Instruments, Phoenix, AZ, USA). Roughness measurements were performed by using a Talysurf CCI Lite white light interferometer (Taylor Hobson, Leicester, UK) with a vertical resolution of 0.01 nm. Basic 3D parameters of surface roughness were determined according to the ISO 25178-2 [37] standard. The profiles were taken perpendicular to the wear track in the middle part of the inner surface of the ribs (Figure 2).

### 2.4. Artificial Neural Networks

The number of neurons in the input and output layers is determined by the number of parameters presented at the input and the number of parameters at the output. The Statistica program was used to analyse the effect of SPIF parameters on the surface roughness value of the stiffening ribs. The ANN algorithm (Intelligent Problem Solver) manuscript was built in the Statistica program, which automatically analyses the training set and possible correlations between input and output parameters for the specified number of multilayer perceptrons *n_a_* = 50. Based on the results of this analysis, the structure of a neural network, which is optimal for the data in the training set, is proposed. The flowchart of the algorithm of the Intelligent Problem Solver module was not discussed in the documentation of the Statistica program. The incremental vertical step size and the tool rotational speed were selected as explanatory parameters. The parameter to be explained was the arithmetical mean height *Sa*, the basic parameter of surface roughness in the machine manufacturing industry [38]. Due to the fact that sheets of various materials and thicknesses were tested, with or without the protective Alclad, it was decided to build an independent network for each of the sheets tested. It is possible to include all these parameters in one global network, but this requires the adding of new neurons at network input and, in consequence, could lead to the loss of the network’s generalisation capabilities due to the limited amount of training data. While the number of neurons in the input and output layers is determined by the number of explanatory and explained variables, there are no general guidelines allowing a priori selection of the number of neurons in the hidden layer(s).

**Figure 3:**
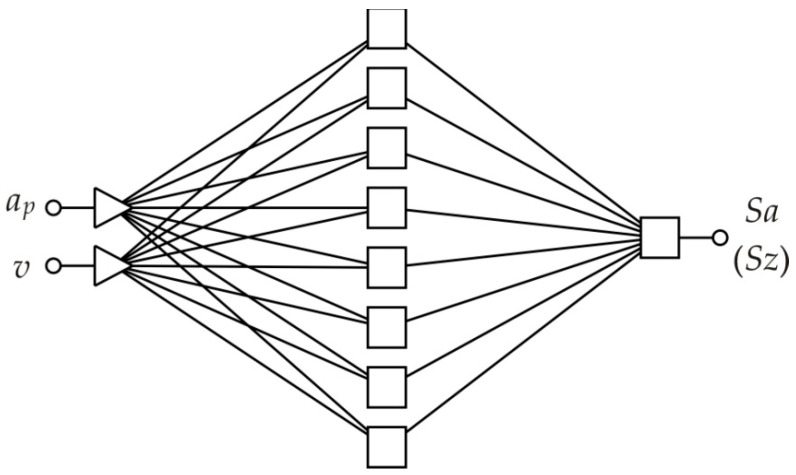
The architecture of the network used to analyse the effect of SPIF parameters on the value of the *Sa* roughness parameter.

An ANN with a 2:2-8-1:1 structure (Figure 3) was selected after conducting a series of analyses with multilayer networks with a different number of neurons in the hidden layer based on the values of two parameters [39,40,41,42], i.e., the coefficient of determination R^2^ (Equation (1)) and round mean square error RMSE (Equation (2)) for the analysis of the influence of SPIF parameters on the value of the average roughness *Sa* and the 10-point peak–valley surface roughness *Sz* of the inner surface of the stiffening ribs. Due to the small size of the training set, this network was used separately to forecast the values of the *Sa* and *Sz* parameters. *Sa* and *Sz* parameters are the most studied parameters of SPIFed surfaces [26]. The network shown in Figure 3 ensured the highest value of the determination coefficient *R^2,^* and, at the same time, the lowest *RMSE* error for data contained in the training set. The values of the network quality parameters were determined as follows:

- Coefficient of determination, *R^2^*:
  (1) $$R^{2}=1-\left(\frac{\sum_{i=1}^{n}{\left(a_{j}-p_{j}\right)}^{2}}{\sum_{i=1}^{n}{\left(p_{j}\right)}^{2}}\right)$$
- Round mean square error, *RMSE*:
  (2) $$RMSE=\sqrt{\frac{1}{n}\sum_{i=1}^{n}{\left|a_{j}-p_{j}\right|}^{2}}$$
  where *a* is the actual value, *p* is the predicted value and *n* is the number of training sets.

The training of the network was carried out with the use of various algorithms, which provided comparable results for the relatively uncomplicated network used in the investigations.

## 3. Results

### 3.1. Surface Topography

**Figure 4:**
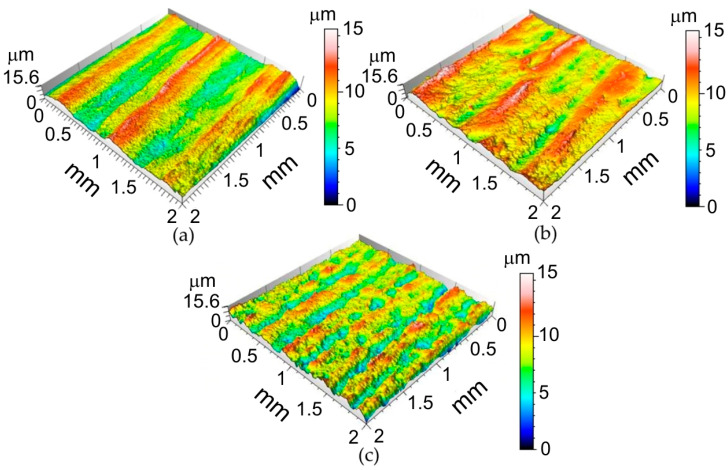
Surface topographies of the inner surface of ribs formed in 1-mm-thick EN AW-2024-T3 fabricated with the following conditions: (**a**) *a_p_* = 0.4 mm, *v* = 18 rpm; (**b**) *a_p_* = 0.4 mm, *v* = 202 rpm; (**c**) *a_p_* = 0.2 mm, *v* = 202 rpm.

**Figure 5:**
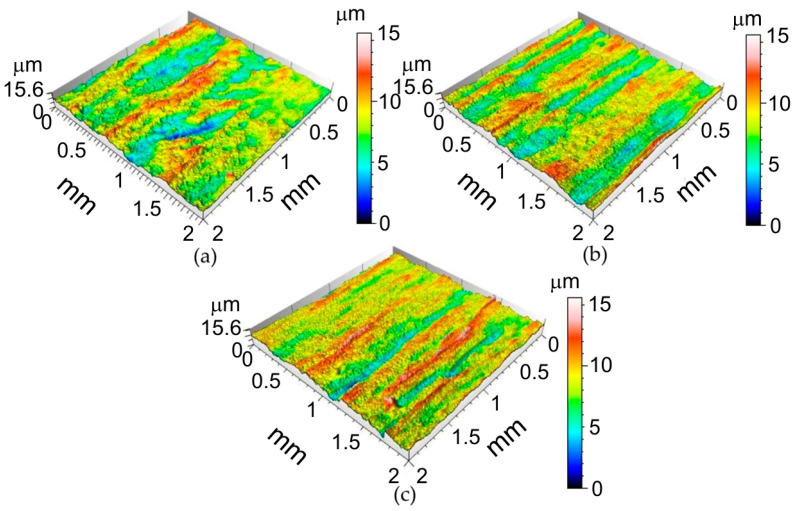
Surface topographies of the inner surface of ribs formed in 0.8-mm-thick EN AW-7075-T6 Alclad fabricated with the following conditions: (**a**) *a_p_* = 0.4 mm, *v* = 18 rpm; (**b**) *a_p_* = 0.4 mm, *v* = 202 rpm; (**c**) *a_p_* = 0.2 mm, *v* = 202 rpm.

**Figure 6:**
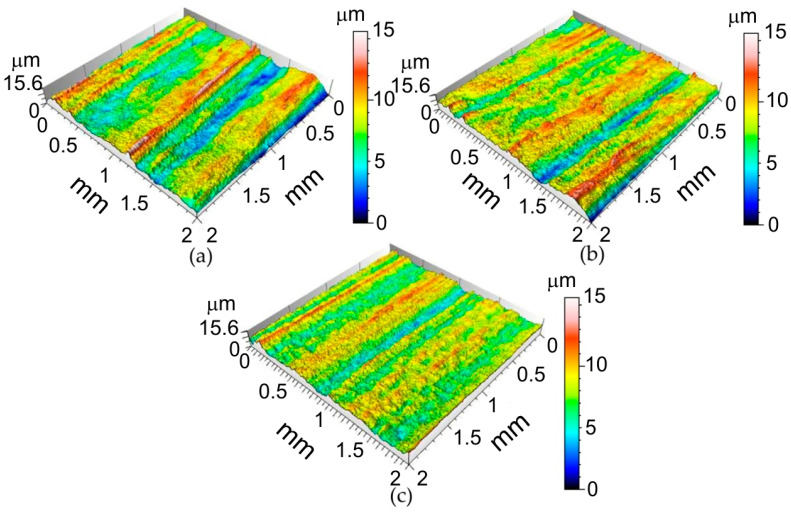
Surface topographies of the inner surface of ribs formed in 0.4-mm-thick EN AW-2024-T3 Alclad fabricated at with the following conditions: (**a**) *a_p_* = 0.4 mm, *v* = 18 rpm; (**b**) *a_p_* = 0.4 mm, *v* = 202 rpm; (**c**) *a_p_* = 0.2 mm, *v* = 202 rpm.

Selected surface topographies and the values of the basic parameters of the stereometric structure of the inner surface of the ribs formed in 1-mm-thick EN AW-2024-T3, 0.4-mm-thick EN AW-2024-T3 Alclad and 0.8-mm-thick EN AW-7075-T6 aluminium alloy sheets are shown in Figure 4, Figure 5 and Figure 6 and Table 5.

It was found that the inner surface of the ribs revealed small linear ridges as a result of the interaction of the spherical-shaped tool tip with the workpiece. The greater the value of the incremental vertical step size, the more prominent the ridges (1-mm-thick EN AW-2024-T3—Figure 4a, 0.8-mm-thick EN AW-7075-T6 Alclad—Figure 5a and 0.4-mm-thick EN AW-2024-T3 Alclad—Figure 6a). Because the difference between the lowest valley and the highest peak on the surface is *Sz*, the tool tip is continuously cultivating the sheet surface and creating new grooves. When the incremental vertical step size *a_p_* is small, then the grooves are continuously plastically deformed, and one can even detect flattening of the surface. The grooves presented in Figure 4b, Figure 5b and Figure 6b correspond to the 1-mm-thick EN AW-2024-T3, 0.8-mm-thick EN AW-7075-T6 Alclad and 0.4-mm-thick EN AW-2024-T3 Alclad, respectively. The asperities on the sheet surface are destroyed and recreated continuously by the topography of the tool. In the case of Alclad sheets (Figure 5 and Figure 6), there is no clear effect of the tool rotational speed on the arrangement and pitch of ridges. However, when forming the EN AW-2024-T3 sheets without Alclad, the highest tool rotational speed caused tearing of the lateral sides of ridges (Figure 4c). A similar effect was observed when forming sheets with Alclad (Figure 5c and Figure 6c). In the case of 1-mm-thick EN AW-2024-T3 sheets, the increase in the vertical step size *a_p_* under constant tool rotational speed leads to an increase in the *Sz* parameter (Table 5). There are no other clear relationships within the specific grade of sheet metal. The relations between *a_p_* and v affecting it are imperceptible due to small differences in the values of parameters. Therefore, it is necessary to build a model that takes the overall view on the change in surface roughness into account. This will be the topic of the next chapter.

### 3.2. ANN Modelling

The training of the network was carried out with the use of various algorithms (quasi-Newton, backpropagation and Levenberg–Marquardt), which provided comparable results for the relatively uncomplicated network used in the investigations. The learning process was characterised by a continuous reduction of the network error along with an increase in the number of epochs, during which the training data were presented to the network. The learning process was carried out until there was no further reduction of the network error [42]. The values of the network for individual learning algorithms and the number of learning epochs necessary to achieve the minimum value of the error function are presented in Table 6. The smallest error values were observed for networks learned with the quasi-Newton algorithm. The number of learning epochs necessary to reach the minimum error value was about 80 for the quasi-Newton algorithm, 140 for the backpropagation algorithm and 170 for the network trained using the Levenberg–Marquardt algorithm.

So, in this paper, the variable metric (quasi-Newton) algorithm was selected to present the results of the training process. The quasi-Newton algorithm ensures the fastest achievement of the error minimum by the network response during the learning process [43,44]. From the training set, 20% of the cases were randomly selected and assigned to the validation set, which is used for independent control of the training algorithm. The validation set protects the learning process against “Occam’s razor”, according to which “in explaining phenomena one should strive for simplicity, choosing such explanations that are based on the fewest assumptions and concepts”. Failure to meet Occam’s condition leads to overfitting of the network to the training data, and, consequently, its ability to generalise the data is lost. *RMSE* error values for the networks are shown in Table 6. The differences in the values of these errors for different networks result from the difference in the possible data noise in the training sets. Another explanation for the differences is the different character of interaction of the spherically ended tool with a clad and an unclad sheet surface. Alclad is an aluminium alloy core material metallurgically bonded to a high-purity aluminium surface layer, which, due to reduced hardness, is much more susceptible to roughening than the core metal. Another explanation for the differences in errors is due to random selection by the learning algorithm of data that were qualified to the validation set. Removal of data that carried significant information from the training set could reduce the quality of the training process.

**Figure 7:**
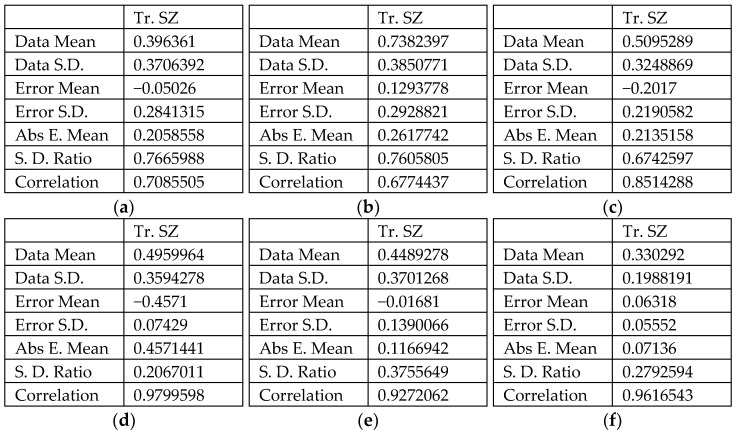
Basic regression parameters of the artificial neural networks modelling the effect of process parameters on roughness parameters *Sa* (**a**–**c**) and *Sz* (**d**–**f**): (**a**,**d**) 0.4-mm-thick EN AW-2024-T3 Alclad, (**b**,**e**) 1-mm-thick EN AW-2024-T3 and (**c**,**f**) 0.8-mm-thick EN AW-7075-T6 Alclad: Data S.D.—standard deviation of data, Error S.D.—error of standard deviation, Abs. E. Mean—absolute error mean, S.D. Ratio—standard deviation ratio, Correlation—coefficient of determination *R*^2^.

The observation of changes in the value of the network learning error is a response to a possible network training error, while next to the network learning error, very important parameters indicating the network’s approximation abilities are the coefficient of determination and the mean of the absolute error. Taking into account the small amount of training data, all networks were characterised by a relatively high value of the coefficient of determination defined for the training set (Tr. SA in Figure 7) above *R*^2^ = 0.657 (Figure 7a–c). Values of the standard deviation ratio above 0.512 for the training set indicate an average quality of the predictive neural network.

The coefficient of determination of neural networks with *Rz* as the explained variable was characterised by a high value of the coefficient of determination *R*^2^ > 0.92 (Figure 7d–f). This means that the input parameters correlate more closely with the roughness parameter *Sz* than with the *Sa* parameter. Moreover, the networks explaining the value of the *Sa* parameter were characterised by an about two times lower value of the standard deviation ratio. The lower the value of this coefficient, the better the quality of the model constructed.

**Figure 8:**
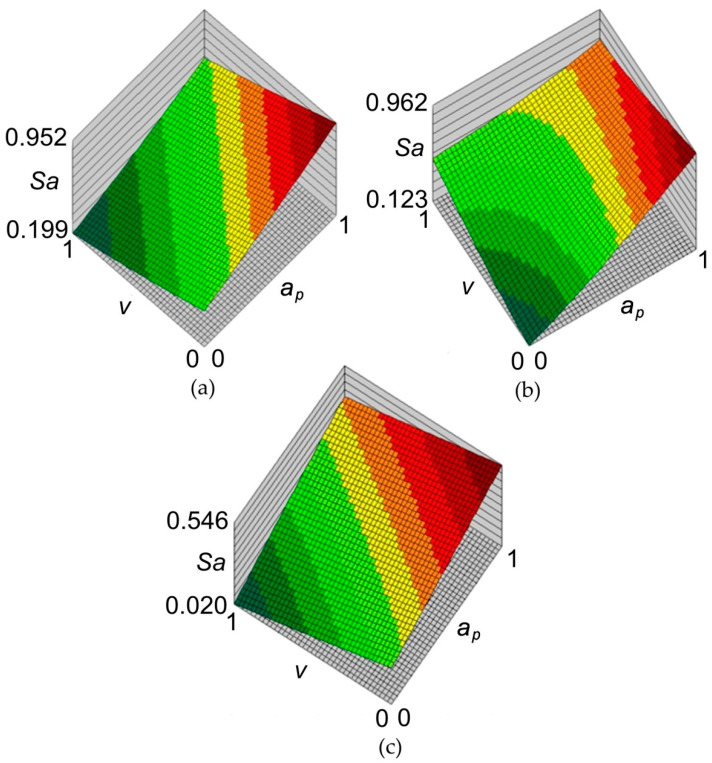
Response surfaces of the neural network 2:2-8-1:1 concerning the influence of incremental vertical step size *a_p_* and the tool rotational speed *v* on the value of the average roughness *Sa* (normalised data) for sheets: (**a**) 0.4-mm-thick EN AW-2024-T3 Alclad, (**b**) 1-mm-thick EN AW-2024-T3 and (**c**) 0.8-mm-thick EN AW-7075-T6 Alclad.

The principal goal of the analyses carried out with the use of neural networks, apart from an attempt to build a high-quality network based on a relatively small set of training data, was to find out the relationship between the parameters of the SPIF process and the value of the average roughness *Sa*. The response surfaces of the neural networks for the data concerning all materials are shown in Figure 8. According to all neural models, increasing the value of the incremental vertical step size ap increases the value of the surface roughness parameter *Sa*. The influence of the tool rotation speed v on the value of the *Sa* parameter is more complex. When forming sheets with an Alclad (Figure 8a,c), increasing the rotational speed *v* reduces the value of the average roughness Sa parameter. A reverse relationship can be observed for the EN AW-2024-T3 sheet (Figure 8b) which did not have a protective anticorrosive coating. This can be explained by the heating of the soft Alclad containing a large proportion of pure aluminium, under the adhesive effect of the surface of the rotating tool. Consequently, the plasticised asperities of the sheet surface were more susceptible to flattening.

**Figure 9:**
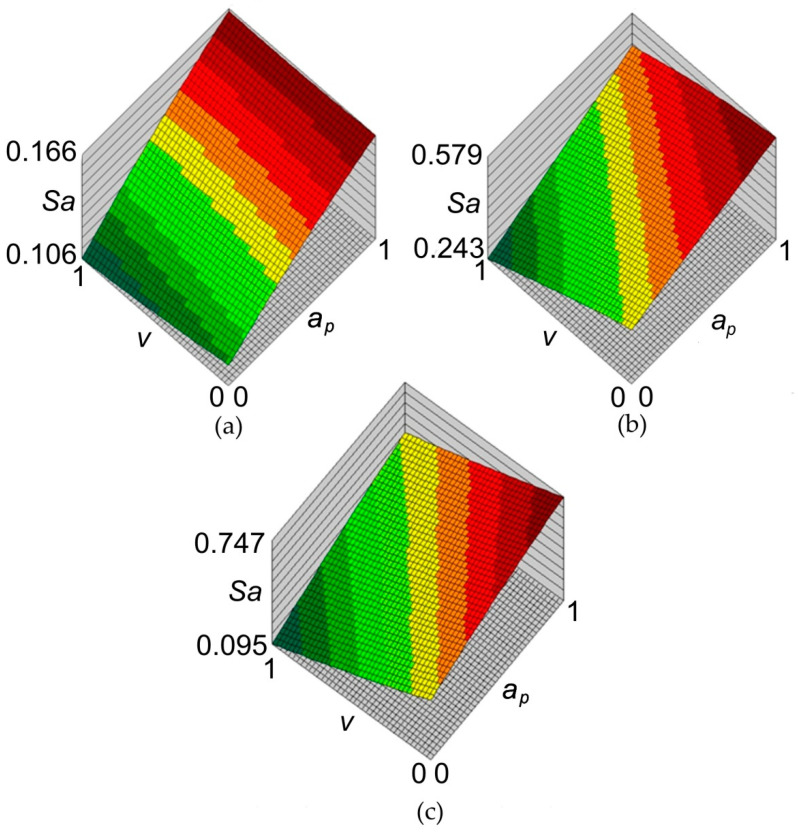
Response surfaces of the neural network 2:2-8-1:1 concerning the influence of incremental vertical step size *a_p_* and the tool rotational speed *v* on the value of the 10-point peak–valley surface roughness *Sz* (normalised data) for sheets: (**a**) 0.4-mm-thick EN AW-2024-T3 Alclad, (**b**) 1-mm-thick EN AW-2024-T3 and (**c**) 0.8-mm-thick EN AW-7075-T6 Alclad.

It was found that during the forming of ribs in Alclad sheets there is a clear relationship between the average roughness *Sa* and the 10-point peak–valley surface roughness *Sz*. Increasing the rotational speed of the tool and vertical step size increases the value of the parameter *Sa* (Figure 9a,c) and the parameter *Sz* (Figure 9a,c). In the case of the sheet not being covered by soft Alclad, the reduction of tool rotational speed increases the *Sz* parameter (Figure 9b) and reduces the *Sa* parameter (Figure 8b). So, the tool rotational speed is a more important parameter in determining the surface finish than incremental vertical step size. In the case of sheets covered by a soft layer of technically pure aluminium, the inner surface of the ribs was more easily ploughed. So, the pronounced directional scratches can be easily correlated with the change of parameters *Sa* and *Sz*. The character of the interaction of the hard surface of the spherically shaped tools with the relatively high-strength of the surface of the greatest thickness (1 mm) of the EN AW-2024-T3 aluminium alloy sheet causes the deformation of the surface asperities. These changes take place in cyclical ridges so the surface asperities are flattened without significant changes in the depths of the valleys.

## 4. Conclusions

In this paper, an analysis is presented on the effect of SPIF parameters on the surface roughness of stiffened ribs formed in EN-2024-T3 and EN AW-7075-T6 aluminium alloy sheets with and without the high-purity aluminium surface layers metallurgically bonded to the core material. The following conclusions are drawn from the experimental research and ANN analyses:

- The greater the value of the incremental vertical step size, the more prominent the ridges found in the inner surface of stiffened ribs, especially in the case of both Alclad aluminium alloy sheets.
- In the case of Alclad sheets, there is no clear effect of the tool rotational speed on the arrangement and pitch of ridges. However, when forming the 1-mm-thick EN AW-2024-T3 sheets (without Alclad), the highest tool rotational speed caused tearing of the lateral sides of the ridges.
- Regardless of the grade of the sheet, an obvious increase in the *Sz* parameter was observed with an increase in the incremental vertical step size.
- The performance measures of the multilayer perceptrons predicting the values of average roughness *Sa* and the 10-point peak–valley surface roughness *Sz* ranges between 0.657 and 0.979, respectively.
- Alclad sheets exhibit a different character of changes of parameters *Sa* and *Sz* from unclad sheets. In the case of Alclad sheets, increasing the value of the incremental vertical step size increases the value of the surface roughness parameters *Sa* and *Sz*. The reduction of the tool rotational speed increases the *Sz* parameter and decreases the *Sa* parameter when forming unclad sheets.

## Figures and Tables

**Table 1:** Chemical composition of test sheets in wt.% [36].

Alloy	Si	Fe	Mn	Cu	Mg	Cr	Zn	Ti	Other Elements		Al
									Each	Total	
2024-T3	0.50	0.50	0.3–0.9	3.8–4.9	1.2–1.8	0.10	0.25	0.15	0.05	0.15	Remainder
7075-T6	0.40	0.50	0.30	1.2–2.0	2.1–2.9	0.18–0.28	5.1–6.1	0.20	0.05	0.15	Remainder

**Table 2:** Basic mechanical properties of test sheets [36].

Material	Temper	Specified Thickness, mm		Tensile Strength *R_m_*, MPa		Yield Stress *R_p_*_0.2_, MPa		Elongation A_50_ min, %	
		Over	Through	min	max	min	max		
EN AW-2024 Alclad	T3	0.25	0.50	405	-	270	-	12	
EN AW-2024	T3	0.50	3.20	435	-	290	-	15	
EN AW-7075 Alclad	T6	0.32	1.00	490	-	420	-	8	

**Table 3:** Basic physicochemical properties of SAE 75W-85 gear oil.

Density *ρ* (at 15 °C), kg/m^3^	Flash Point, °C	Pour Point, °C	Viscosity at 40 °C, mm^2^/s	Viscosity Index
879	210	−45	72.4	157

**Table 4:** Orthogonal array design.

Number of Experiment	Incremental Vertical Step Size *a_p_*, mm	Tool Rotational Speed *v*, rpm
E1	0.2	18
E2	0.2	110
E3	0.2	202
E4	0.3	18
E5	0.3	110
E6	0.3	202
E7	0.4	18
E8	0.4	110
E9	0.4	202

**Table 5:** Basic amplitude parameters of the surface roughness of the ribs formed in 1-mm-thick EN AW-2024-T3 sheets.

*a_p_*, mm	*v*, rpm	*Sq*, μm	*Ssk*	*Sku*	*Sp*, μm	*Sv*, μm	*Sz*, μm	*Sa*, μm
0.2	18	1.09	0.355	3.10	5.16	4.33	9.49	0.874
	110	0.94	0.0009	2.73	3.85	3.69	7.54	0.733
	202	0.99	−0.0872	2.60	4.05	4.30	8.36	0.799
0.3	18	1.16	−0.321	3.37	3.86	4.67	8.53	0.914
	110	1.53	0.0228	2.45	5.09	4.77	9.86	1.26
	202	0.93	−0.184	3.50	3.34	4.91	8.25	0.737
0.4	18	1.92	0.192	2.65	7.12	8.51	15.6	1.59
	110	1.19	0.0649	3.35	4.93	6.65	11.6	0.940
	202	1.39	−0.0958	3.69	4.88	8.29	13.2	1.09

**Table 6:** *RMSE*s for the 2:2-8-1:1 networks trained using different algorithms.

Sheet Material	Output Variable	*RMSE* for Training Set		
		Quasi-Newton	Back Propagation	Levenberg–Marquardt
0.4-mm-thick EN AW-2024-T3 Alclad	*Sa*	0.1206	0.1278	0.1381
	*Sz*	0.4624	0.4821	0.4997
0.8-mm-thick EN AW-7075-T6 Alclad	*Sa*	0.3004	0.3189	0.3047
	*Sz*	0.0817	0.0974	0.1086
1-mm-thick EN AW-2024-T3	*Sa*	0.2860	0.3068	0.3091
	*Sz*	0.1311	0.1457	0.1531

## Data Availability

The data presented in this study are available on request from the corresponding author.
